# The Use of Serum Glial Fibrillary Acidic Protein Measurements in the Diagnosis of Neuromyelitis Optica Spectrum Optic Neuritis

**DOI:** 10.1371/journal.pone.0023489

**Published:** 2011-08-18

**Authors:** Mithu Storoni, Axel Petzold, Gordon T. Plant

**Affiliations:** 1 The National Hospital for Neurology and Neurosurgery, London, United Kingdom; 2 Moorfields Eye Hospital, London, United Kingdom; 3 Department of Neurology, VU Medisch Centrum, Amsterdam, The Netherlands; National Institutes of Health, United States of America

## Abstract

**Background:**

Glial fibrillary acidic protein (GFAP) is a specific intermediate filament of the cytoskeleton of the astrocyte and may be used as a specific marker for astrocytic damage. It is detectable in the cerebrospinal fluid following a relapse caused by Multiple Sclerosis (MS) and Neuromyelitis Optica (NMO) spectrum disease. Higher levels are found following an NMO-related relapse. It is not known if GFAP is also detectable in the serum following such relapses. In particular, it is not known if lesions limited to the optic nerve release GFAP in sufficient quantities to be detectable within the serum. The aim of this study was to ascertain the extent to which serum GFAP levels can distinguish between an episode of optic neuritis (ON) related to NMO spectrum disease and ON from other causes.

**Methodology/Principal Findings:**

Out of 150 patients consecutively presenting to our eye hospital over the period March 2009 until July 2010, we were able to collect a serum sample from 12 patients who had presented with MS-related ON and from 10 patients who had presented with NMO spectrum disease-related ON. We also identified 8 patients with recurrent isolated ON and 8 patients with a corticosteroid-dependent optic neuropathy in the absence of any identified aetiology. GFAP was detectable in the serum of all but three patients (two patients with MS-related ON and one with recurrent optic neuritis). The median serum GFAP level in the patient group with NMO spectrum disease was 4.63 pg/mL whereas in all other cases combined together, this was 2.14 pg/mL. The difference was statistically significant (P = 0.01). A similar statistically significant difference was found when cases with pathology limited to the optic nerve were compared (P = 0.03).

**Conclusions:**

Glial pathology in NMO related optic neuritis is reflected in elevated serum GFAP levels independently of whether or not there is extra-optic nerve disease.

## Introduction

The conclusions of the optic neuritis treatment trial (ONTT) have guided the management of isolated idiopathic optic neuritis (ON) around the world [1]. The ONTT suggested that both no treatment and intra-venous corticosteroid therapy were equally satisfactory treatment options in the context of typical ON.

Neuromyelitis Optica (NMO) has emerged as a separate disease entity even in cases presenting with ON without myelitis, following the identification of the antibody to Aquaporin 4 [2], [3]. There may be a time lag between the ON episode and the ensuing myelitis [2], [4]. Although patients with NMO related ON may present with a more severe episode of ON than seen in multiple sclerosis (MS), there is little else to distinguish one type of ON from another at first presentation. Following the ONTT treatment protocol in NMO related ON patients (and offering no treatment) may cause preventable nerve fibre loss [5]. Furthermore, offering subsequent MS immunomodulatory treatments to NMO patients may be harmful [6].

A small cohort of patients present with acute, isolated ON who do not have the Aquaporin 4 autoantibody, and who follow a different course to the majority of cases described in the ONTT. Some, within this category, require corticosteroids for recovery and continued immunosuppression for the prevention of further attacks of ON, whilst others may have repeated attacks of ON which resolve without treatment. These patients display this ‘atypical’ pattern without showing any evidence of MS on imaging [7], [8]. Some authors have since classified these patients within syndromic groups such as recurrent isolated optic neuritis (‘RION’) and chronic relapsing inflammatory optic neuropathy (‘CRION’) [8], [9].

In western Europe, the majority of acute ON cases encountered resemble the clinical profile described in the ONTT, and hence require no treatment [10]. However, for those patients with isolated ON at first presentation who do need immediate treatment, early identification would be very useful, and may be sight-saving [5].

The discovery of the NMO specific autoantibody to the water channel Aquaporin 4 (found extensively on astrocytic foot processes) provided evidence for the consideration of NMO as a separate disease entity from MS.

Recent research has focused on the detection of markers of astrocytic damage, and their relative expression in NMO and MS. Glial fibrillary acidic protein (GFAP) is a specific intermediate filament (IF) of the cytoskeleton of the astrocyte and is absent from oligodendrocytes and neurons. GFAP measured in cerebrospinal fluid (CSF) has been shown to be marginally increased in MS patients when compared with normal controls but substantially increased in NMO patients undergoing relapses [11]–[14].

It has until now not been shown if GFAP may be measured in the serum following an MS or NMO spectrum relapse, especially if the relapse involves a single lesion in the optic nerve. It is also not known if ‘atypical’ patterns of ON (such as recurrent or corticosteroid-dependent: ‘RION’ and ‘CRION’) result in differing degrees of astrocytic damage.

This is the first study to our knowledge, to investigate levels of ***serum*** GFAP in the context of isolated ON. We asked three main questions.

1. Is it is possible to detect GFAP in the serum of patients with ON associated with MS and NMO spectrum disease and is there a difference in the serum level between these two groups? It has been shown previously that other biomakers (nitric oxide and neurofilaments) can be detected in serum following an episode of optic neuritis even though the volume of diseased tissue is relatively small and levels are likely to be lower than in CSF [15].
2. Does isolated optic nerve disease in the absence of lesions elsewhere result in the release of sufficient levels of GFAP into the serum, to be detectable?
3. Do the levels of serum GFAP (and hence the degree of astrocytic damage) found in patients with ‘atypical’ presentations of ON, such as ‘CRION’ and ‘RION’ pattern ON, resemble MS or NMO, or are they distinct from both? A significant difference between ‘CRION’ and MS patients would allow the former to be identified earlier and given urgent immunotherapy.

We also investigate if the level of serum GFAP is related to the degree of recovery from the most recent attack of ON. We show that it is possible to measure GFAP in the serum of optic nerve patients and that this measurement may allow us to identify NMO from non-NMO related ON.

## Methods

### Objectives

1. To investigate if NMO Spectrum ON can be distinguished from MS associated ON by measuring serum GFAP following an episode of ON, even in the absence of extra-optic nerve lesions.

2. To investigate if serum GFAP levels in patients with ‘atypical’ patterns of ON (such as ‘CRION’ or ‘RION’ pattern ON) resemble those found in patients with NMO spectrum ON.

### Participants

Out of 150 patients consecutively presenting to our eye hospital over the period March 2009 until July 2010 with an episode of acute ON at Moorfields Eye Hospital or The National Hospital of Neurology and Neurosurgery, Queen Square, we were able to collect a serum sample (within 210 days of onset of the ON episode) from 12 patients who had presented with MS related ON and from 10 patients who had presented with NMO spectrum disease-related ON. MS was diagnosed based on the McDonald criteria [16]. The NMO spectrum group were all positive for the Aquaporin 4 antibody, 6 patients presented with isolated ON only and 4 patients also had myelitis at some point in the past or concurrently and hence satisfied Wingerchuk's criteria for diagnosis [17]. We also identified 8 patients who had suffered from ON which relapsed upon the withdrawal of steroid therapy, in whom MR imaging the optic nerve, brain and spinal cord did not show demyelination (ie, any lesion in addition to the optic neuropathy), and there had been at least 3 relapses in the absence of any other evidence for disease including sarcoidosis, based on imaging and serology. We labeled this group as ‘CRION’ [9]. In a fourth group, 8 patients had experienced at least two episodes of ON. 5 of these patients were treated with a short course of steroid therapy at their own request, and did not relapse upon its withdrawal. The patients displayed no evidence of demyelination elsewhere. As they displayed a recurrent pattern of ON in the absence of evidence of other pathology, we labeled this group as ‘RION’ [8]. All patients except for those with MS related ON were tested for the Aquaporin 4 antibody. All patients with other existing neurological/connective tissue/ vasculitic/ ophthalmic disease were excluded from the study.

The demographic data of the participants are given in Table 1.

**Table 1:** The age, gender and ethnic background of patients across all categories are shown.

	ON Subtype^1^			
	MS related ON	NMO spectrum	‘CRION’ pattern ON	‘RION’ pattern ON
White Caucasian ethnic background	6	2	3	6
Not ‘white Caucasian’ ethnic background	6	8	7	2
F∶M	8∶3	7∶3	9∶1	5∶3
Median Age in years (s.d.)^2^	32 (11)	34 (10)	44 (10)	42 (13)

A higher female: male ratio is observed in all groups. The majority of patients with NMO spectrum and ‘CRION’ pattern ON do not have a ‘white Caucasian’ ethnic background.

1 ON = optic neuritis, NMO = Neuromyelitis Optica, MS = Multiple Sclerosis, ‘CRION’ = ‘chronic relapsing inflammatory optic neuropathy’, ‘RION’ = Recurrent Isolated Optic Neuritis.

2 s.d. = standard deviation.

### Patient Groups

#### MS group

The MS group was made up of 12 patients. In 4 of these, the ON episode was the first episode of MS which was diagnosed subsequently. Four of the 8 patients in whom the diagnosis was already confirmed at the point of sample collection had experienced previous non-optic nerve relapses. None of the participants were on long term immunomodulatory therapy.

#### NMO spectrum group

This group was formed of 10 patients. All patients were seropositive for the antibody to Aquaporin 4. Of these, 6 patients had suffered from ON only, without any evidence of myelitis; these patients will be referred to as ‘AQP4+ON’. The remaining 4 patients satisfied Wingerchuk's criteria [17].

#### ‘RION’ group

This group consisted of 8 patients. Two of the patients presented with their first attack during the time of the study. None of the participants were on long term treatment of any kind.

#### ‘CRION’ group

This group included 8 patients, all of whom had been diagnosed prior to the current episode of ON, and all of whom were on some form of immunosuppression at the time of the attack.

### Serum Analysis for GFAP level

Serum samples were collected in polypropylene tubes centrifuged (2,000 g for 10 minutes) and stored immediately at −80°C in 1.5–2 mL Eppendorf tubes (polypropylene) until analysis. Serum GFAP was measured in duplicates with the analyst being blinded to all other information using an in-house developed ELISA [18]. The analytical accuracy of the assay was found to be 5.8% (interassay coefficient of variation).

### Aquaporin 4

All patients were tested for Serum aquaporin 4 antibodies (AQP4) aside from those with a diagnosis of MS associated ON. Testing was carried out at the Wetherall Institute of Molecular Medicine, University of Oxford by a method using the fluorescence immunoprecipitation assay (FIPA) technique described elsewhere [19].

### Measurement of visual recovery

All patients were clinically assessed using a Snellen chart. We defined visual recovery in terms of the number of lines of improvement using a Snellen chart, from the point of the worst visual acuity during the acute episode to the visual acuity measured at a follow up assessment after recovery. In the case of bilateral optic neuritis, we used the reading from the eye with the greater degree of recovery. For comparison of the visual acuity during the peak of visual loss during an episode of ON, we converted the Snellen reading to a LogMar measurement for ease of comparison of visual acuities across the groups.

### Ethics

Informed written consent was obtained from all patients. The Central London Research Ethics Committee granted ethical permission for the study (REC reference number: 09/H0716/63). The study was conducted in accordance with the principles expressed in the Declaration of Helsinki.

### Statistical methods

SigmaPlot© and SigmaStat© statistical software were used to perform statistical analysis on the data. Tukey Box Plots were used to graphically represent the data showing either the 5^th^, 25^th^, 50^th^, 75^th^ and 95^th^ percentiles, or the 25^th^, 50^th^, and 75^th^ percentiles. The student t-test was used for the comparison of two samples if the samples followed a normal distribution. Where this was not the case, the Mann–Whitney Rank Sum test was used instead. The Kruskal-Wallis One Way Analysis of Variance was used to compare multiple groups. Multinear regression analysis was used to assess the influence of more than one independent factors on a dependent factor. A p-value of 0.05 was accepted as significant.

## Results

**Figure 1:**
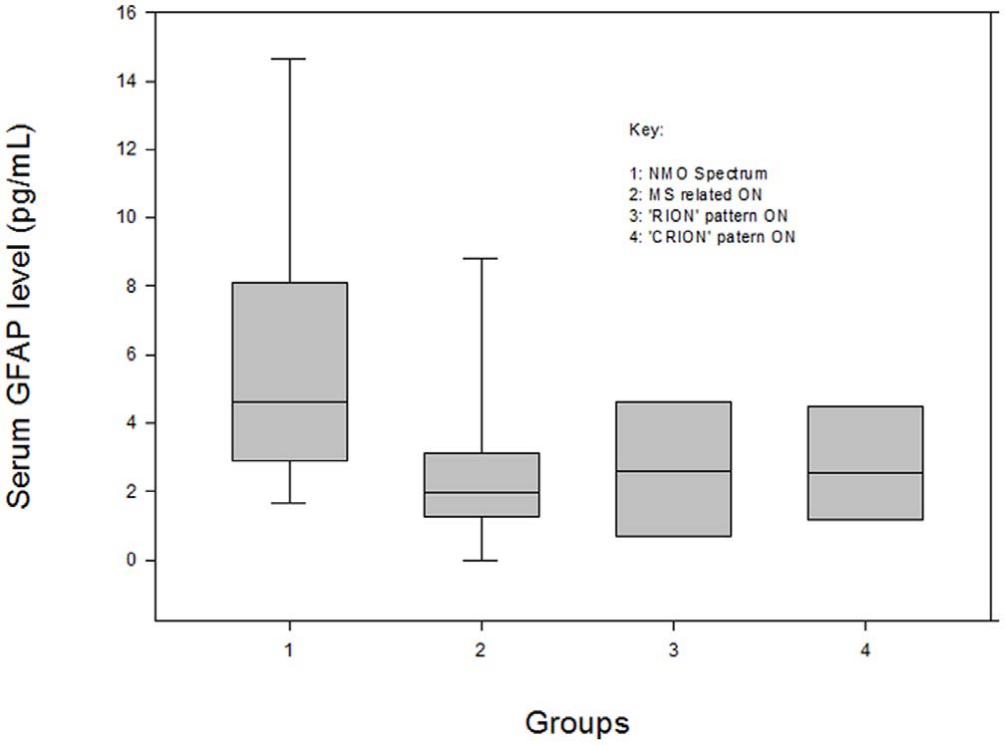
Serum GFAP Level (pg/mL) Across All Groups, Shown in Tukey Box Plot. Tukey box plot showing the median, 25th percentile and 75th percentile of serum GFAP level measurements in each group. The 5th and 95th percentile of groups 1 and 2 are also shown.

The serum GFAP levels in pg/mL measured in patients with NMO spectrum disease, MS-related ON, and ‘CRION’ and ‘RION’ syndromes are represented in Figure 1. Both the mean and median values of serum GFAP demonstrate a trend in the following order: NMO>‘CRION’>‘RION’>MS.

**Figure 2:**
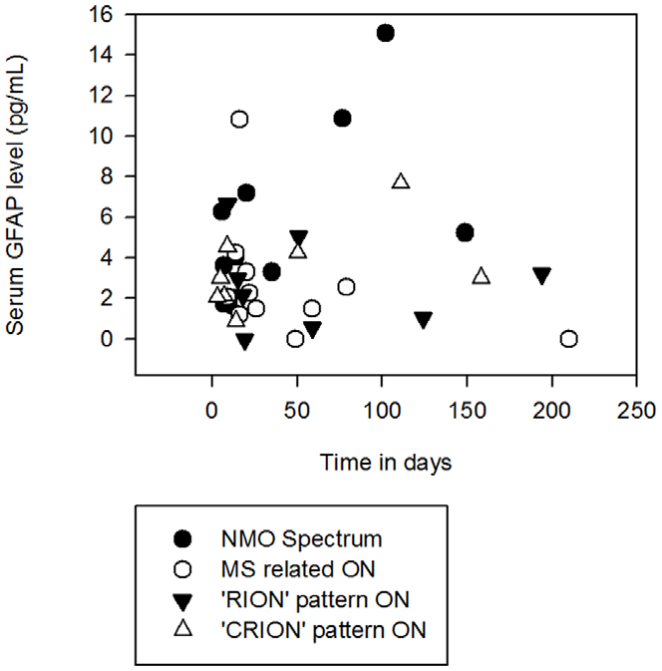
Time in Days Following the Onset of Acute Optic Neuritis, When Serum GFAP Measurements Were Acquired, Versus the Serum GFAP Level in pg/mL. Although many patients' serum was tested within 50 days of onset, their serum samples were collected up to 210 days following the onset of acute optic neuritis.

The serum GFAP level was measured after variable time intervals (until 210 days) following the acute optic neuritis episode onset. Figure 2 demonstrates the time interval (in days) after which the serum GFAP was measured in all patient groups, and the serum GFAP level.

### 1) Serum GFAP levels in NMO vs MS and in NMO vs all AQP4 negative cases

A comparison of the NMO Spectrum group with the MS group is made in Table 2. The Mann-Whitney Rank Sum Test confirmed a statistically significant difference (P = 0.02) between the median value of the NMO spectrum group (4.63 pg/mL) and the median value of the MS group (1.96 pg/mL). 75% of readings within the NMO spectrum group fell above 3.3 pg/mL whereas 75% of readings within the MS group fell below 2.94 pg/mL and the 99% confidence interval of the NMO spectrum group was 4.36 pg/mL, whereas that of the MS group was 2.56 pg/mL.The MS group showed an excess kurtosis of 7.0 compared to 1.3 in the NMO group.

**Table 2:** Descriptive statistics of serum GFAP measurements (pg/mL) in patients across all categories^3^ are shown.

	NMO (pg/mL)	MS related ON (pg/mL)	‘RION’ (pg/mL)	CRION’ (pg/mL)	All patients with non-NMO spectrum ON
Mean	5.90	2.60	2.71	3.16	2.79
Std Dev^1^	4.25	2.85	2.28	2.29	2.46
C.I. of Mean^2^	3.04	1.81	1.91	1.91	0.96
Median	4.63	1.96	2.58	2.56	2.14
25%	3.30	1.33	0.80	1.46	1.11
75%	7.21	2.94	4.16	4.39	3.77
Skewness	1.29	2.42	0.62	1.08	1.60
Kurtosis	1.30	6.99	−0.45	1.12	3.16
99% C.I.^2^	4.36	2.56	2.82	2.83	

The fifth column shows the merged data of all ‘non-NMO spectrum’ patients. Both mean and median values for the serum GFAP level were highest in the NMO spectrum category and lowest in the MS related ON category. The results from patients with ‘atypical’ patterns of optic neuritis (‘RION’ and ‘CRION’ pattern) fell halfway between the two groups. Patients in the NMO spectrum group showed the highest variance. All values are stated to within 2 decimal places.

1 Std Dev = standard deviation.

2 C.I. = Confidence Interval.

3 ON = optic neuritis, AQP4+ON = Aquaporin 4 autoantibody positive ON, ‘CRION’ = ‘chronic relapsing inflammatory optic neuropathy’, ‘RION’ = Recurrent Isolated Optic Neuritis.

The NMO Spectrum group is compared with all other patients (MS, ‘RION’ syndrome and ‘CRION’ syndrome combined together) in Table 2. The Mann-Whitney Rank Sum Test confirmed a statistically significant difference (P = 0.01) between the median value of the NMO spectrum group (4.63 pg/mL) and the median value of the three other groups combined together (2.14 pg/mL). 75% of readings within the NMO spectrum group fell above 3.30 pg/mL whereas 75% of readings within the three other groups, when combined, fell below 3.77 pg/mL.

The Mann-Whitney Rank Sum Test did not show a statistically significant difference between the median values of the MS and ‘CRION’ groups (P = 0.44), nor between the median values of the NMO and ‘CRION’ groups (P = 0.17).

Serum GFAP is statistically significantly higher in NMO spectrum patients than in patients with MS, as well as all Aquaporin 4 antibody negative patients combined together.

### 2) Serum GFAP levels in AQP4 positive patients with isolated optic neuritis vs. all AQP4 negative cases

The question arises as to whether it is the occurrence of extra-optic nerve disease – such as myelitis – that is the factor determining the higher GFAP levels in NMO.

**Figure 3:**
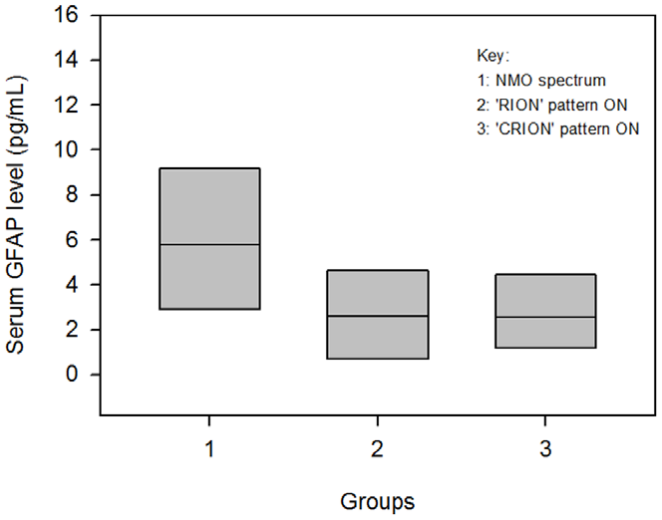
Serum GFAP Level (pg/mL) Measurements in Patients without Extra-Optic Nerve Disease (AQP4+ON, ‘RION’ and ‘CRION’ Groups). Tukey box plot showing the median, 25th percentile and 75th percentile of serum GFAP level measurements in each group.

The serum GFAP levels (in pg/mL) measured in patients without extra-optic nerve disease (AQP4+ON, ‘CRION’ syndrome and ‘RION’ syndrome) are shown in Figure 3. The descriptive statistics for the comparison of AQP4+ON patients with all antibody negative patients who do not have extra-optic nerve disease (‘RION’ syndrome and ‘CRION’ syndrome) are shown in Table 3. Use of the t-test confirmed a statistically significant difference (P = 0.03) between the mean value of the AQP4+ON group (6.46 pg/mL) and the mean value of the ‘RION’ and ‘CRION’ syndrome groups combined (2.94 pg/mL). The 99% confidence interval of the AQP4+ON group was 7.70 pg/mL whereas those of the ‘RION’ and ‘CRION’ groups were 2.82 pg/mL and 2.83 pg/mL respectively.

**Table 3:** Descriptive statistics of serum GFAP measurements (pg/mL) in patients without optic nerve disease^1^ are shown.

	NMO (pg/mL)	‘RION’ (pg/mL)	‘CRION’ (pg/mL)	R+C (pg/mL)
Mean	6.46	2.71	3.16	2.94
Std Dev^1^	4.68	2.28	2.29	2.22
C.I. of Mean^2^	4.91	1.91	1.91	1.18
Median	5.76	2.58	2.56	2.58
25%	3.30	0.80	1.46	0.95
75%	7.21	4.16	4.39	4.39
Skewness	1.45	0.62	1.08	0.75
Kurtosis	2.72	−0.45	1.12	−0.09
99% C.I.^3^	7.70	2.82	2.83	

AQP4+ON patients showed the highest levels of serum GFAP as well as the highest variance. Both the median and mean values for serum GFAP level are higher in AQP4+ON patients compared to the values in the NMO spectrum group as a whole (4.63 pg/mL, 5.90 pg/mL). All values are stated to within 2 decimal places.

1 Std Dev = standard deviation.

2 C.I. = Confidence Interval.

3 ON = optic neuritis, AQP4+ON = Aquaporin 4 autoantibody positive ON, ‘CRION’ = ‘chronic relapsing inflammatory optic neuropathy’, ‘RION’ = Recurrent Isolated Optic Neuritis.

Serum GFAP is statistically significantly higher in Aquaporin 4 antibody positive patients without extra-optic nerve disease than in all Aquaporin 4 antibody negative patients combined together.

### 3) Serum GFAP levels and visual outcome

This analysis was carried out with 9 NMO spectrum patients, 9 MS patients and 7 ‘RION’ patients on whom we had accurate data on visual acuity. The worst visual acuity during the most recent episode of acute ON (baseline visual acuity, measured as a LogMar conversion of the Snellen reading) was not significantly different between the groups. A Kruskal-Wallis Analysis showed there was no statistically significant difference between the groups (P = 0.30) and the Mann-Whitney Rank Sum Test confirmed the absence of a statistically significant difference between the median baseline visual acuity of the NMO spectrum group (1.0) and that of the MS and ‘RION’ syndrome groups (0.8), when combined. The Mann-Whitney Rank Sum Test also confirmed the absence of a statistically significant difference between the median baseline visual acuity of the NMO spectrum group (1.0) and that of the MS group (0.8).

Multiple linear regression analysis did not demonstrate a correlation between the number of lines (on a Snellen chart) of recovery following optic neuritis and the serum GFAP level measured, taking into account the number of days following the optic neuritis episode, when the serum measurement was undertaken.

## Discussion

This study has shown that Glial pathology in NMO related optic neuritis is reflected in elevated serum GFAP levels independently of whether or not there is extra-optic nerve disease. The level of serum GFAP during an attack of ON has no prognostic value.

Our results suggest it may be possible to separate patients with NMO spectrum disease from those with MS when they present with an episode of acute ON, on the basis of their group means. A larger number of cases would be required in order to determine the sensitivity and specificity of the test in individual cases, as the difference in serum GFAP levels was not great enough to prevent some overlap in values across the groups. The significant difference in the level of serum GFAP found in this study nonetheless holds promise for the use of serum GFAP to predict whether a previously healthy patient presenting for the first time is likely to require a non-ONTT based treatment protocol.

The differences in the level of serum GFAP between patients in the groups we have studied were less dramatic than those reported in a recent study examining levels of GFAP in the cerebrospinal fluid of patients with NMO and MS, where the overall levels of cerebrospinal fluid GFAP measured during a relapse were reported to be 2 476.6±8,815.0 ng/mL in NMO and 0.8±0.4 ng/mL in MS [12]. However, a relapse in the form of optic neuritis was found to result in markedly lower levels of cerebrospinal GFAP (median 6.1; range 1.1–56.1) than myelitis (median 593.9; range 1.2–47,843.3 ng/mL).

All our patients experienced a relapse in the form of optic neuritis. Additionally, measurement of GFAP in the serum instead of the cerebrospinal fluid is likely to result in a lower measurement following passage across the blood-brain-barrier and may explain the difference in the levels reported between the two studies. Takano et al (2010) carried out cerebrospinal fluid measurements in almost half of all patients within a week of onset and all patients within 25 days of onset and demonstrated a sharp decline in GFAP level (a factor of 100 within 25 days) over this time. In contrast, serum analysis in our study was carried out in fewer than half of all patients within 14 days and in all patients up to 210 days following onset. This may be an additional reason behind the difference in GFAP levels detected in the two studies.

We did not have sufficient numbers of patients to make a comparison between patients presenting for the first time with what is later diagnosed as NMO spectrum disease, versus patients presenting for the first time with what later becomes MSON.

The observation that isolated astrocytic damage in the optic nerves is sufficient to release GFAP at concentrations quantifiable from the serum, and that these are highest in NMO spectrum ON further supports a role for serum GFAP in the diagnosis of NMO spectrum ON at first presentation.

The absence of a statistically significant difference between the serum GFAP values in the ‘CRION’ pattern ON group and either the MS or NMO spectrum groups may be due to the small sample size, or may be a consequence of immunosuppression. Most patients with ‘CRION’ were immunosuppressed prior to the most recent episode of ON and this immunosuppression may have protected against the full extent of inflammation occurring during the attack, and limited astrocytic damage.

As our testing methods for the autoantibody to Aquaporin 4 have been shown to be 76% sensitive and 100% specific, there exists a possibility that the ‘RION’ and ‘CRION’ cohorts within this study also contain NMO spectrum patients which may have reduced the differences in serum GFAP levels between the groups [19]. A previous study has described cases of ‘RION’ pattern ON developing into NMO spectrum disease over time [2]. Our study provides some evidence that the astrocytic damage occurring within the ‘RION’ pattern of ON is less extensive than that occurring in AQP4+ON, and that patients with a ‘RION’ pattern of ON may instead have a similar extent of astrocytic damage to MS associated ON. Although all patients labeled as having a ‘RION’ pattern of ON in our study showed no demyelination on imaging, the overlap between a ‘RION’ pattern of optic neuritis and MS has been described [20].

Patients from the AQP4+ON, MSON and ‘RION’ groups suffered from statistically similar degrees of visual loss during the acute episode of ON (P = 0.30). In all three groups, no correlation of the level of visual recovery following the episode was found with the serum GFAP level. Although this may be the result of testing small sample sizes and the wide temporal window within which sampling was carried out (210 days), an additional reason may be that visual outcome reflects neuronal loss, not astrocytic damage. Serum GFAP may be released as a result of astrocytic damage without resulting neuronal loss, for which other neuron-specific markers such as neurofilaments have been shown to be better biomarkers [15].

We were unable to measure the level of serum GFAP over time within individual patients and this is a significant limitation of our study. The rate of decay of serum GFAP level following astrocyte damage is not known.

Serum GFAP levels need to be measured in patients presenting with acute isolated ON for the first time, with no known medical history, in order to accurately determine the role of GFAP in the diagnosis of optic neuritis.

The number of participants was small and a larger cohort would be required to reach further conclusions. This would require a longer time period of observation in a long-term prospective study.
